# Post-transplant diabetes mellitus

**DOI:** 10.1186/1758-5996-1-14

**Published:** 2009-10-05

**Authors:** Marília B Gomes, Roberta A Cobas

**Affiliations:** 1 Diabetes and Metabology Unit from Pedro Ernesto University Hospital, Medical Science School, State University of Rio de Janeiro (UERJ; 2 Program in Clinical and Experimental Pathophysiology (PGCLINEX) Diabetes and Metabology Unit, Pedro Ernesto University Hospital, State University of Rio de Janeiro (UERJ

## Abstract

In recent decades, Diabetes Mellitus has become a severe and growing global public healthcare problem due to the increase of its prevalence, morbidity and mortality. Post-transplant diabetes mellitus (PTDM) is a complication which takes place after a solid organ transplant, and its incidence is widely variable, ranging from 2 to 53%. Some factors increase the risk of PTDM, such as age, ethnicity, cadaver-donor kidney presence of the hepatitis C virus and cytomegalovirus, overweight and obesity and the Immunosuppression scheme established in the immediate post-transplant period. High doses of tacrolimus and corticosteroid represent the highest risk for developing PTDM.

Considering that the development of PTDM is associated with a higher risk of complications, such as infections and cardiovascular disease - thus representing a higher life threatening risk and a higher cost for the Health System - the relevance of identifying the risk factors and of the early diagnosis combined with appropriate therapy will be high for the follow up, and eventually resulting in the success of the procedure as far as patient survival and transplantation durability.

## Introduction

In recent decades, Diabetes Mellitus has become a severe and growing global public health problem in developed and developing countries due to the increase of its prevalence, morbidity and mortality. Recent estimates by the World Health Organization (WHO) forecast a significant increase in the number of individuals suffering from diabetes until the year 2030. Then, the number of estimated diabetes-suffering individuals constitutes a universe of nearly 366 million people [1]. Approximately 90% of them will develop type 2 Diabetes Mellitus (T2DM), in the age range of 45-64 years-old, in developing countries, where it is known that the access conditions to specialized medical centers are not always satisfactory [1].

Post-transplant diabetes (PTDM) is a complication that occurs after a solid organ transplant, being also considered as a secondary type of diabetes mellitus [2]. The very first cases were described in 1964 after a liver transplant by Thomas Starzl. The latest estimates about its incidence report a wide variability, of 2-53%, of which 4-25% occurred after a kidney transplant and 2.5-25% after a liver transplant. Such variability of post-transplant diabetes is reportedly related to the difficulty in defining, diagnosing and identifying the potential risk factors associated with this entity [3]. As for post kidney transplant diabetes, estimating that the number of chronic kidney disease patients under dialysis treatment in the world is around 1.5 million, we may infer that the number of post kidney transplant diabetes cases will increase significantly in the next few decades [4]. The same assumption may also apply to other solid organ transplants.

Considering that the development of post-transplant diabetes is associated with a higher risk of complications, such as infections and cardiovascular disease - thus representing a higher life-threatening risk and a higher cost for the Healthcare System - the relevance of identifying risk factors and of the early diagnosis combined with appropriate therapy will be high for the 4 follow-up, and eventually resulting in the success of the procedure as far as patient survival and transplant long-term durability [3,5-10].

This review aims at discussing and establishing some procedures to facilitate the approach to those patients in the daily clinical practice.

### 1. Identifying Risk Factors

Some of the factors that increase the risk of developing post-transplant diabetes have already been identified. Instructively, such factors could be assorted in non-modifiable, potentially modifiable and modifiable risk factors (table 1). Here will be only considered the potentially modifiable and the modifiable risk factors. In the potentially modifiable group, cytomegalovirus infection (both asymptomatic and symptomatic) is the most prevalent (which may alter the secretion of insulin [6] and the hepatitis C virus (HCV) infection which is more widely associated with insulin resistance, though it may cause a cytopathic effect on beta cells [11,12]. The treatment using interferon in the pre-transplant period for HCV patients significantly reduced the incidence of post-transplant diabetes [2]. The presence of IGT in the pre-transplant period is already a condition requiring lifestyle changes [5,6].

**Table 1:** Risk factors for post-transplant diabetes development

**Non-modifiable**	**Potentially modifiable**	**Modifiable**
Ethnicity (non-Caucasian)	Infections	Immunosuppressive Therapy
Age >40 years-old	• HCV	• Tacrolimus
Recipient's gender (M)	• CMV	• Cyclosporine
Donor's gender (M)	IGT (pre-transplantation)	• Corticosteroid
Family history of DM		• Sirolimus
HLA		Obesity
HLA (mismatches)		MS components
Cadaver-donor		
History of acute rejection		

IGT, Impaired glucose tolerance; MS, Metabolic Syndrome

The modifiable risk factors include corticosteroid therapy that increases the resistance to peripheral and hepatic insulin and calcineurin inhibitors (cyclosporine e tacrolimus), that cause further reduction of insulin secretion by a direct toxic effect on beta cell [13-18]. Despite differences in doses of PTDM doses and definition criteria, the use of tracolimus is generally associated with a risk of developing PTDM 30% higher than cyclosporine, that presents an 18% risk [19-22], mainly in HCV patients [20]. Some studies recommend that the maximum plasma concentration of tracolimus in the immediate post-transplant period be 15 ng/mL[2]. For patients taking tracolimus and cyclosporine, the incidence peak of IGT and/or diabetes was 60 days after the transplant; however, at 6 and 12 months, the renal PTDM incidence was still higher in the group treated with tracolimus, as compared to the group treated with cyclosporine [14]. The ideal dose of corticoid so as not to induce a dramatic increase in PTDM is still under discussion. In a study conducted in our environment, the prednisone dose >1.3 mg/kg/day was associated with a higher risk of renal PTDM. The use of low prednisolone doses, 5 mg/day, seems to be the most indicated [3].

Regarding the presence of obesity and other SM components, the most appropriate action would be the early lifestyle change (diet + physical exercises) still in the pre-transplant period, according to the patient's clinical condition and control over other risk factors, such as hypertension and dyslipidemia [5].

### 2. Diagnosis of glucose intolerance and diabetes

According to the latest International Consensus about PTDM, every patient in the pre-transplant period must be examined for glucose intolerance and diabetes. The anamnesis and clinical history of the patient will also be important for the identification of risk factors and co-morbidities.

The criteria for diagnosing glucose intolerance and post-transplant diabetes follow the standards established by the American Diabetes Association (ADA) [23] and Brazilian Society of Diabetes Association (SBD) [24] as described below:

#### 2.1 Diabetes

Diabetes symptoms with randomized plasma blood glucose ≥200 mg/dL (11.1 mmol/L) or

Fasting plasma glucose (FPG) (at least 8 hours fast) ≥126 mg/dL (7.0 mmol/L)

#### 2.2 Fasting intolerance

FPG ≥110 mg/dL (6.1 mmol/L) and < 126 mg/dL (7.0 mmol/L)

2.3 Oral test for glucose intolerance (glucose load at 75 g of anhydrous glucose dissolved in water) 2-hour plasma glucose ≥140 mg/dL (7.8 mmol/L) and < 200 mg/dL (11.1 mmol/L)

The diagnosis of any glucose intolerance must be confirmed by a test on the following day.

### 3. Clinical management of patients with PTDM

The clinical managing of patients with PTDM is generally the same as recommended for T2DM and as established by ADA, Brazilian Society of Diabetes (SBD) and other guidelines [23-27].

#### 3.1 Glucose and risk factors control

Desired Glucose level: HbA1c <6.5%

LDL-cholesterol <100 mg/dL(<2.59 mmol/L)

HDL-cholesterol >50 mg/dL (1.3 mmol/L) for women and >40 mg/dL (1.0 mmol/L) for men

Triglycerides <200 mg/dL (2.6 mmol/L)

Systolic blood pressure <130 mm Hg and diastolic blood pressure <80 mm Hg

Body weight control

#### 3.2 Therapeutical approach

In case diet and physical exercise are not enough to reach the desired glucose and lipid levels, diabetes treatment must include oral drugs, combined oral therapy and also insulin and even insulin monotherapy (it will be required for 25% of the patients). Regarding oral agent therapy, the following items should be observed:

1. Metformin: assess kidney function for the risk of lactic acidosis

2. Sulfonylurea: drugs metabolized and eliminated through the kidney may cause hypoglycemia mainly in elderly patients. Glinide would not have such effects.

3. Glitazones: for acting on the insulin resistance, they could be indicated for these patients, but the side effects must be assessed (weight gain, edema, anemia, pulmonary edema and heart failure). The risk of fractures must be considered, mainly in patients that make chronic use of corticosteroid.

4. GLP-1 analog and DPP-IV inhibitors: there is no information around the use of these drugs for PTDM. Both GLP-1 and GIP incretins are eliminated through the kidneys.

5. Immunosuppression individualization: to assess the replacement of tracolimus for cyclosporine and the use of low corticosteroid doses

Regarding dyslipidemia, it is considered that the treatment using statin must be established if the proper LDL level is not reached. The blood pressure treatment must be thorough and the renal function must be monitored. Drug interactions must be carefully assessed. Drugs metabolized by cytochrome P-450 isoenzyme CYP 3 A4 must be monitored. Inducers (rifampicin, carbamazepine, phenytoin) and inhibitors (cyclosporine, gemfibrozil) of that system may modify the kinetics of some oral agents such as repaglinide, increasing its half-life and resulting in hypoglycemia.

### 4. Follow-up for PTDM patients

Recommendations: quarterly HbA1c determination, lipid profile twice or thrice a year, microalbuminuria screening, yearly ophthalmologic analysis, feet examination on every follow-up visit.

## Conclusion

Patients diagnosed with PTDM have higher risk of cardiovascular disease and infections than the general population and such problems may compromise the survival period and the transplant durability [6,7]. PTDM is considered a significant cause of morbidity e mortality in transplant patients. The early identification of such condition in addition to a thorough treatment of diabetes and its co-morbidities will definitely determine its progression.

## Competing interests

The authors declare that they have no competing interests.

## Authors' contributions

MBG: has written the manuscript

RAC: has revised the literature

All authors read and approved the final manuscript.
